# Point Cloud Semantic Segmentation Network Based on Multi-Scale Feature Fusion

**DOI:** 10.3390/s21051625

**Published:** 2021-02-26

**Authors:** Jing Du, Zuning Jiang, Shangfeng Huang, Zongyue Wang, Jinhe Su, Songjian Su, Yundong Wu, Guorong Cai

**Affiliations:** 1 Computer Engineering College, Jimei University, Xiamen 361021, China; jingdu@jmu.edu.cn (J.D.); jzn201721121073@gmail.com (Z.J.); shangfenghuang@jmu.edu.cn (S.H.); wangzongyue@jmu.edu.cn (Z.W.); sujh@jmu.edu.cn (J.S.); yundongwu@jmu.edu.cn (Y.W.); 2 Ropeok Technology Group Co., Ltd., Xiamen 361021, China; songjian.su@ropeok.com; 3 Fujian Collaborative Innovation Center for Big Data Applications in Governments, Fuzhou 350003, China

**Keywords:** LIDAR point cloud, semantic segmentation, feature fusion, deep learning, computer vision

## Abstract

The semantic segmentation of small objects in point clouds is currently one of the most demanding tasks in photogrammetry and remote sensing applications. Multi-resolution feature extraction and fusion can significantly enhance the ability of object classification and segmentation, so it is widely used in the image field. For this motivation, we propose a point cloud semantic segmentation network based on multi-scale feature fusion (MSSCN) to aggregate the feature of a point cloud with different densities and improve the performance of semantic segmentation. In our method, random downsampling is first applied to obtain point clouds of different densities. A Spatial Aggregation Net (SAN) is then employed as the backbone network to extract local features from these point clouds, followed by concatenation of the extracted feature descriptors at different scales. Finally, a loss function is used to combine the different semantic information from point clouds of different densities for network optimization. Experiments were conducted on the S3DIS and ScanNet datasets, and our MSSCN achieved accuracies of 89.80% and 86.3%, respectively, on these datasets. Our method showed better performance than the recent methods PointNet, PointNet++, PointCNN, PointSIFT, and SAN.

## 1. Introduction

Deep learning algorithms have achieved significant success in many remote sensing image analysis tasks, including object detection, semantic segmentation and classification. On the one hand, the purpose of semantic segmentation is to assign a land cover label to each pixel in an image. Facilitated by deep convolutional neural networks (CNNs), especially end-to-end fully convolutional networks (FCN) [1], interest in the semantic segmentation of remote sensing images has increased in recent years. Furthermore, semantic segmentation focusing on the detection of small objects in remote sensing images [2,3,4,5,6] and in point clouds covering global navigation satellite system (GNSS) indoor and underground environments [7] has become a very attractive research topic.

In the research of a 3D point cloud, semantic segmentation is a hot research topic in the field of autonomous driving and robot localization. Segmentation algorithms that take input in the form of point clouds can be roughly divided into three categories: multiview-based [8,9,10,11,12,13], voxel-based [14,15,16,17,18], and raw-point-cloud-based algorithms [19,20,21,22,23,24,25,26,27]. The transformation of point clouds to a regular 3D voxel or images usually leads to serious loss of geometric information and increases the calculation complexity. Therefore, algorithms based on an original point cloud have become a hot research field recently. The original point cloud contains rich geometric and semantic information, so it is easier for algorithms to realize scene perception. Typical algorithms include PointNet [19], PointNet++ [20], PointCNN [28], and PointSIFT [29]. Although the point-based deep learning models have made remarkable progress in the past three years, they still face difficulties related to the avoidance of information loss in the process of down-sampling. Objects with fewer points will keep fewer points in the final sampled points. Different densities of classes will increase the difficulty of segmentation.

In the field of image, multi-resolution feature extraction and fusion [30] can significantly enhance the ability of object classification and segmentation. Motivated by this phenomenon, we propose a point cloud semantic segmentation network based on multi-scale feature fusion, which can aggregate features of different densities and improve the performance of semantic segmentation. Firstly, point clouds of different densities are obtained by changing the sampling ratio. Low-density point clouds in the proposed network are suitable for extracting global shape features of a target, while high-density point clouds are suitable for extracting detail from local features. Then, features are extracted from point clouds of different scales. Finally, a new feature set is obtained from the extracted features by applying the feature fusion operation. To sum up, there are three main contributions in our work:

Firstly, we propose a multi-scale feature fusion architecture that is suitable for point clouds. The multi-scale point cloud is obtained via stepwise downsampling from the same original point cloud. We set different sampling ratios for different datasets and achieve promising segmentation accuracy compared to state-of-the-art methods on both the ScanNet and Stanford Large-Scale 3D Indoor Spaces (S3DIS) datasets.

Secondly, our MSSCN fuses point features extracted from different network levels through direct mapping and concatenation. This feature fusion method not only allows the advantages of the feature representations extracted at each level to be combined, but also avoids error propagation at each level.

Finally, we design a loss function for MSSCN, which is used to train the network by combining losses at different scales. Experimental results demonstrate that each component of the loss function influences the final segmentation accuracy of MSSCN.

The rest of the paper is organized as follows. In the Section 2, the literature on point cloud segmentation and classification is reviewed. Section 3 introduces the proposed deep learning network structure MSSCN in detail. In the Section 4, the experimental setup is introduced and the results are discussed. The Section 5 is a summary of the paper.

## 2. Related Work

Point clouds do not have a regular structure, whereas the input data for traditional CNNs [31,32,33,34] must have a regular format; consequently, traditional CNNs are not suitable for extracting the features of a point cloud. Previously, researchers have been working to transform 3D point clouds into regular formats that are similar to images or voxels. For example, in the multi-view based methods [35], the original point cloud is projected into the image plane based on its depth or intensity values, and the projection views are generated from a virtual camera posture. A typical example of this approach is MVCNN [8]. Since 2018, projection-based methods have been widely concerned. For instance, the Pointwise Rotation-Invariant Network [36] framework was proposed to achieve rotation invariance in point clouds. RotationNet [37] is effective for real scenes, because it only uses a part of the original multi-view images to perform the inference process. However, due to the loss of local geometry during the compression of 3D data to 2D data, methods based on the projection of point clouds still face some limitations. Ref. [38] uses multiple clues to integrate range and color content, in order to retain local geometric information. In this context, Ref. [39] maps an input point cloud to a scanning pattern grid. Virtual MVFusion [40] provided additional channels to render virtual views, which exceeds the limitations of existing RGB-D sensors. At the same time, Virtual MVFusion designed the backside culling scheme and multi-scale view sensing sampling. Therefore, the occlusion, narrow view and scale invariance problems that plagued most previous multi-view fusion methods were improved.

Voxel, as small units of points set in 3D space, can be used to divide a point cloud into a regular 3D subspace. Most voxel-based deep learning architectures are inspired by 2D CNNs. Generally, 0–1 discrete values are used to confirm whether there are any points in a specific voxel. As a typical method of this type, 3D ShapeNet [15] employed binary voxels for three-dimensional filtering. However, this scheme always leads to an increase in computational complexity. Therefore, researchers are attempting to improve the network structure of voxel CNNs, as in [41,42].

Recently, researchers have paid increasing attention to semantic segmentation networks that take the original point cloud as the initial input. In this case, the input vector for the deep neural network can be composed of coordinates or a combination of coordinates, intensity, and color information. For algorithms based on raw point clouds, it is necessary to solve the problem of achieving invariance in the order of input points. The representative method for settling this conundrum is PointNet [19]. This networks use global feature pooling to make the output vector invariant to the sequence of the input point. However, it is difficult to extract local geometric features for each point since max-pooling layers can be applied only to all points. To effectively overcome this challenge, PointNet++ [20] used a multilevel network structure for the extraction of local features. That being said, the max-pooling operation is also adopted in PointNet++. As a consequence, the network only retains the maximum feature feedback from global and local regions, resulting in a loss of useful geometric information that adversely affects the segmentation task. PointCNN [28] used different levels of representative points to realize the feature extraction schemes proceeding from local regions to the global point cloud. However, this method may introduce a new problem. In most cases, the distribution of the point cloud is uneven, which will lead the selected representative points to gather in a small space. Consequently, after several convolution operations, the reception range will be limited. To handle this problem, PointSIFT [29] selected points adjacent to the representative points in a specified direction. The purpose is to acquire a complete description of the spatial structure features around key points. The disadvantage of PointSIFT is the relatively high time complexity. Recently, [43] designed the novel PointConv operation to achieve network expansion and improve segmentation performance. Ref. [44] proposed a multi-directional convolutional network—called a Spatial Aggregation Network (SAN)—which can utilize local spatial structure information to achieve relatively high efficiency and accuracy.

However, due to the complexity of the point cloud distribution, the features of the chosen alternative points may not be representative of the original features. In this situation, the geometric information for each point will be ignored, which may lead to the loss of local feature information. To extract local geometric and global features synchronously, the authors of TGNet [45] proposed a novel convolution filter that extracts point features in a hierarchical and multiscale manner. Experimental results showed that this strategy effectively combines features from different scales and improves the performance of local region segmentation. The authors of DGCNN [46] proposed an innovative edge convolution that can extract the geometric features of local neighborhoods while maintaining permutation invariance. However, this edge-based convolution obtains neighborhood points based only on distance, which may still lead to local geometric information loss. GeoCNN [47] extracted features based on the angle aggregation between edge vectors and orthogonal bases, so as to keep the geometric structure in the whole feature extraction process. However, it is worth noting that GeoCNN needs to recalculate the *K* nearest neighbors for all points in each stage, resulting in higher complexity. KPConv [48] can be expanded to deformable convolutions by adapting the kernels to local geometries. Any number of kernel points can be used, giving KPConv more flexibility than grid convolutions. Furthermore, these locations are spatially continuous and can be learned by the network. A new convolution operator learned from relationships in RS-CNN [49] is called relational shape convolution, which can encode the geometric relationship of points and expand the configuration of regular grid CNNs to achieve context-aware learning of point clouds. FPConv [50] proposed the surface style convolution operator. The operator disperses the convolution weight of each point along the local surface, so it is robust to input data. Finally, points are projected onto the 2D grid by predicting projection weights, and regular 2D convolution can be used for feature learning. However, CNNs may not correctly solve the problem of non-Euclidean data. Therefore, graph convolutional networks (GCNs) were developed to overcome this challenge by creating graphs representing non-Euclidean data. With the help of techniques for increasing the depth of CNNs, DeepGCNs [27] were developed using not only residual/dense connections but also dilated convolutions. Residual/dense connections can solve the problem of gradient disappearance caused by an increase of network depth. By expanding the convolution kernel to increase the receptive field without increasing the number of parameters, the dilated convolutions help to solve the problem of spatial information loss caused by pooling. Finally, a 56-layer GCN was constructed in this way, which offered significantly improved performance in the semantic segmentation of point cloud. Grid-GCN [51] proposed Coverage Aware Grid Query (CAGQ), which samples representative center points and queries adjacent points. CAGQ implements data structuring and makes full use of grid space efficiency, thus increasing space coverage and reducing theoretical time complexity. CAGQ is up to 50 times faster than the most popular sampling methods such as farthest point sampling and spherical query. A Graphic Convolution Module Grid Context Aggregation (GCA) is proposed to integrate context features and coverage information into computation. SPVNAS [52] proposed Sparse Point-Voxel Convolution (SPVConv), which is equipped with a high-resolution point-based branch for sparse convolution. It then introduced the first 3D Neural Architecture Search (3D-NAS) for 3D scene understanding, a framework that searches for the optimal network structure within a given resource constraint. JSENet [53] introduced semantic edge detection into semantic segmentation. Semantic edge detection provides detailed edge location information and can generate accurate edges. At the same time, dual semantic edge loss is proposed to improve the segmentation effect of the edge position.

Algorithms based on raw point clouds [20,28,29,43] typically require several downsampling operations. In this scheme, objects with fewer points will keep fewer points at the final sampled points. Different density of categories will increase the difficulty of segmentation [27,45,46,47,48,49].

## 3. The Proposed Approach

**Figure 1:**
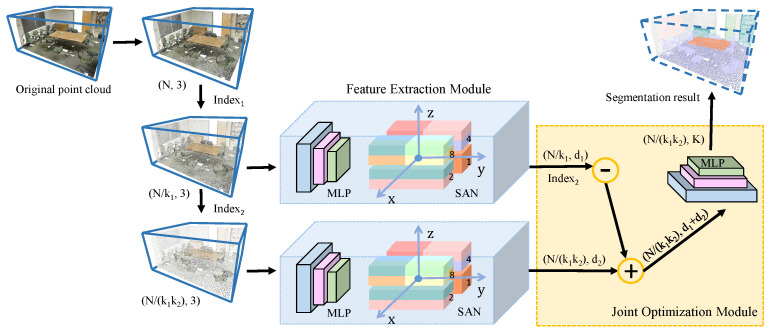
Illustration of the proposed multi-scale feature fusion network (MSSCN). First, downsampling is performed on the original point cloud with sampling proportions of $k_{1}$ and $k_{2}$. The chosen points are stored in $Index_{1}$ and $Index_{2}$ for the first and second downsampling processes, respectively. Then, feature extraction is performed using a Spatial Aggregation Net (SAN) backbone, where $d_{1}$ and $d_{2}$ are the dimensionalities of the features for each downsampled point cloud. Finally, feature fusion is performed to obtain a relevant set of features, where ‘−’ indicates the deletion of descriptors that do not exist in $Index_{1}$ according to $Index_{2}$ and ‘+’ indicates feature fusion. Based on the extracted features, a multilayer perceptron (MLP) is used to obtain the score of each point for each of the *K* object categories.

The network structure of MSSCN is shown in Figure 1. First, downsampling is applied to obtain the point cloud. Then, we extract features from the point clouds at each scale using the network architecture proposed in our previous work, SAN. Finally, feature fusion and optimization are performed on the extracted features.

### 3.1. Multiscale Point Feature Extraction

To make our method invariant to scale changes, a multi-scale point feature extraction method based on different densities is proposed. We find that features of low-density point clouds are suitable to represent global shape features, while high-density point clouds are appropriate to describe detailed local features. According to the characteristics of the point clouds, features associated with different densities are complementary. To construct the multi-scale feature fusion network, we perform random downsampling operations on the input data. Specifically, three scales are used to construct point clouds via downsampling. Thus, we obtain two point clouds $P_{1}$ and $P_{2}$ of different densities, representing the point clouds after the first and second downsampling processes, respectively. We record the position of each sampled point in the two down-sampling point clouds, which can be used for feature fusion later. The selection of the sampling ratios for datasets with different characteristics is discussed in Section 4.1.

Then, we extract features of each point from the multi-scale point clouds. Since SAN achieves an effective balance between efficiency and accuracy, it is deemed a good backbone network for abstracting features from $P_{1}$ and $P_{2}$. In particular, SAN uses a hierarchical structure that combines small area features into semantic features. It contains not only several Directional Spatial Aggregation (DSA) components but also some feature unencoding (FP) modules. The DSA module is the core module of SAN. It is divided into three steps for extracting the features of sampling points. First, point downsampling is performed using the farthest point sampling (FPS) [54]. Secondly, the neighboring points around each sampling point are captured by octant search [29]. Finally, the multi-directional convolution operation is performed on the sampling points. This convolution is followed by max pooling to aggregate features from different directions. The point cloud $P_{1}$ contains $N/k_{1}$ points. The SAN network extracts features for each point in $P_{1}$, and the feature dimension of each point is $d_{1}$. The point cloud $P_{2}$ contains $N/(k_{1}k_{2})$ points, and the SAN network extracts $d_{2}$ dimension features for each point in $P_{2}$.

### 3.2. Feature Fusion and Loss Function

**Figure 2:**
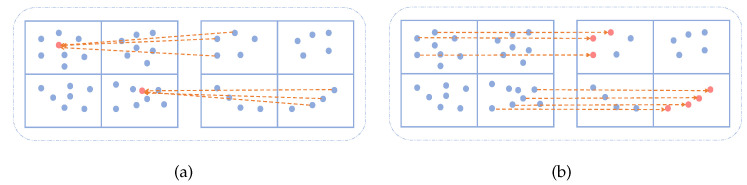
Feature fusion process: (**a**) feature interpolation based on distance and (**b**) direct mapping.

Many methods can be used to fuse features from point clouds of different densities, such as descriptor interpolation based on the distances between adjacent points, as shown in Figure 2a. In this method, the information of one point is combined with information from its neighboring points, and weighted according to distance. However, two points belonging to different classes may be aggregated using this method, which leads to unstable segmentation. Figure 2b shows a direct mapping method for feature fusion, which aims to alleviate this problem. As shown in Equations (1) and (2). $F_{1}$ and $F_{2}$ represent the feature descriptors of the points of $P_{1}$ and $P_{2}$, respectively. $Index_{1}$ is the index set of the points in $P_{1}$. $Index_{2}$ is an index set of points in $P_{2}$. Feature fusion combines the point feature $F_{1}$ of $P_{1}$ with the feature $F_{2}$ from $P_{2}$. The process of feature fusion is divided into two steps. First, we delete the points not in $Index_{2}$ from $P_{1}$ according to $Index_{2}$, so that $P_{1}$ and $P_{2}$ retain the same points. At this time, the indexes of the points of $P_{1}$ and $P_{2}$ are both $Index_{2}$. But because SAN extracts features from point clouds of different densities, the features are not the same. Therefore, the feature descriptor corresponding to the point of $P_{1}$ is re-expressed as $F_{2}^{{}^{\prime }}$. In the second step, we perform feature fusion through the consistency of the point index. By concating the point features with the same index in $P_{1}$ and $P_{2}$, we obtain a set of features with $d_{1}+d_{2}$ dimensions, named $F_{3}$. Finally, the score of each point in *K* categories is obtained through the Multilayer Perception (MLP) operation.

(1) $$\begin{array}{c} F_{2}^{{}^{\prime }}=F_{1}\left[Index_{1}-Index_{2}\right],F_{2}^{{}^{\prime }}\in R^{d_{1}} \end{array}$$

(2) $$\begin{array}{c} F_{3}=F_{2}⊕F_{2}^{{}^{\prime }},F_{3}\in R^{d_{1}+d_{2}} \end{array}$$

**Figure 3:**
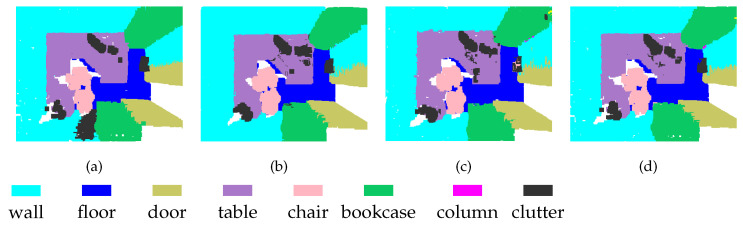
Segmentation results of point clouds with different densities: (**a**) ground truth; (**b**) point cloud at Level-1 ($P_{1}$), where the sampling proportion is 1/2; (**c**) point cloud at Level-2 ($P_{2}$), where the sampling proportion is 1/4; and (**d**) MSSCN.

Because we maintain the point index correlations in the process of downsampling, feature fusion via direct mapping will not lead to redundant calculation. However, the direct mapping scheme has some disadvantages, one of which is that not all points in the high-density set have multi-scale descriptors. To work around this problem, the sampling rate was set to be greater than or equal to 1/2 in our experiments. Therefore, the lowest density of $P_{1}$ was half the original density, as shown in Figure 3b, while the lowest density of $P_{2}$ was 1/4 the original density, as illustrated in Figure 3c. We applied SAN extraction to the point clouds at each scale. The corresponding segmentation results from redlevel 1 and level 2 are presented in Figure 3b,c, respectively. Although the densities of the point clouds are different, the segmentation results are stable. Figure 3d shows the segmentation results of MSSCN.

We also present a loss function that incorporates a different loss for each density to increase the robustness of MSSCN for multiscale point cloud scenes. The loss function is shown in Equation (3). Equations (4)–(6) are annotations for each variable in the loss function. The loss function has four components. $\alpha_{1},\alpha_{2},\alpha_{3}$, and $\alpha_{4}$ are parameters that determine the trade-off among the four components. The first and second components, respectively, use the cross-entropy loss $L_{seg}$ to penalize the wrong segment labels in the level 1 and level 2 predictions. $L_{seg}$ denotes the cross-entropy classification loss. The third component punishes points with incorrect segmentation labels in the final predictions. $pre_{1}$, $pre_{2}$, and $pre_{3}$ represent prediction results. $label_{1}$, $label_{2}$, and $label_{3}$ represent ground truth labels. In an ideal environment, the predictions obtained for $P_{2}$ using $F_{2}$ and $F_{2}^{{}^{\prime }}$ should be consistent. Therefore, the fourth component is used to enhance the consistency of the predictions using $F_{2}$ and $F_{2}^{{}^{\prime }}$. $Index_{1}$ represents the indexes of points downsampled from the primitive input data. $Index_{2}$ denotes the indexes of points obtained via the second downsampling process from $P_{1}$. *N* is the number of points in the original point cloud. $S_{1}$ is the ratio of the first down-sampling, and $S_{2}$ is the ratio of the second down-sampling. The loss function is shown as follows:

(3) $$\begin{array}{cc} Loss & =\alpha_{1}L_{seg}\left(pre_{1},label_{1}\right)+\alpha_{2}L_{seg}\left(pre_{2},label_{2}\right)+\\ \; & \alpha_{3}L_{seg}\left(pre,label_{2}\right)+\alpha_{4}\frac{1}{N*S_{1}*S_{2}}\sum (0.5\\ \; & *\left(pre_{2}!=label_{2}\right)+0.5*\left(pre_{2}^{{}^{\prime }}!=label_{2}\right)) \end{array}$$

(4) $$pre_{2[i]}^{{}^{\prime }}=pre_{1[Index_{2}\left[i\right]]},i=0,...,N*S_{1}*S_{2}$$

(5) $$label_{1[i]}=label_{\left[Index_{1}\left[i\right]\right]},i=0,...,N*S_{1}$$

(6) $$label_{2[i]}=label_{1[Index_{2}\left[i\right]]},i=0,...,N*S_{1}*S_{2}$$

### 3.3. Algorithm Summary

The pipeline of our proposed MSSCN method is shown in Algorithm 1. The input to the network is a point cloud scene, where each point is associated with three-dimensional coordinate information (x,y,z), denoted by *P*. The other information on the points in *P* is recorded as the feature set *F* of those points. *N* is the number of points in the point cloud. The output of the network is the score of each point for each class. First, we perform downsampling on the input data. Specifically, $N\times S_{1}$ indexes, denoted by $Index_{1}$, are randomly generated in the range [0,N) without duplicates. Similarly, $N\times S_{1}\times S_{2}$ indexes are randomly generated in the range $\left[0,N\times S_{1}\right)$, which are denoted by $Index_{2}$. Here, $S_{1}$ and $S_{2}$ are the proportions used in the first and second downsampling processes, respectively. In the second step, using $Index_{1}$ as the indexes of the points in *P*, a new point cloud $P_{1}$ is generated. Similarly, $Index_{2}$ is used as the indexes to generate a new point cloud $P_{2}$. The features of these point clouds of different densities are then extracted by the SAN feature extractor in the third step.

SAN uses the FPS algorithm to obtain a new point cloud $P_{new}$. For each point $P_{i}$ in $P_{new}$, the adjacent 3D space centered on $P_{i}$ is divided into eight octants. SAN selects the $\frac{k}{8}$ nearest points as the representative points in each octant. In the experiments described in the following section, to ensure that there would be four points in each direction. We set the initial value of *k* to 32. Then, a feature vector fusion operation is employed for all points in the same direction, using a convolution operator to fuse the feature vectors of the four points into a single vector. Next, we use 2×1 convolution operators to aggregate the points from all eight directions into only four directions. The convolved features representing the spatial structure information of each point are obtained through this multi-directional convolution. An MLP is used to transform the new features, and finally, the seven features are grouped using the max-pooling operation to obtain a new feature set $F_{new}$ corresponding to $P_{new}$. Then, we repeat the above operation three times to obtain $P_{new1}$ and $F_{new1}$, $P_{new2}$ and $F_{new2}$, $P_{new3}$ and $F_{new3}$, and we weight the corresponding features based on distance. Finally, the newly acquired features are merged with the original features. In detail, the feature set $F_{new3}$ of $P_{new3}$ is mapped to $P_{new2}$, the feature set $F_{new2}$ of $P_{new2}$ is mapped to $P_{new1}$, the feature set $F_{new1}$ of $P_{new1}$ is mapped to $P_{new}$, and the feature set $F_{new}$ of $P_{new}$ is mapped to $P_{1}$. Finally, the feature set $F_{1}$ corresponding to $P_{1}$ is obtained. The feature set $F_{2}$ corresponding to $P_{2}$ is also obtained in this manner.

Then, feature fusion is performed as shown in steps 4 and 5. We use $Index_{2}$ to obtain the points in $P_{1}$, then obtain the new feature set $F_{2}^{{}^{\prime }}$ of these points and combine the feature set $F_{2}^{{}^{\prime }}$ of $P_{2}$ with the new feature set $F_{2}^{{}^{\prime }}$ to obtain feature set $F_{3}$ for $P_{2}$. Finally, the MLP operation is performed on $F_{3}$ to classify every point in $P_{2}$, and the result is recorded as $P_{seg}$. The proposed MSSCN presents many advantages. At first, MSSCN extracts features using SAN. In addition, with the development of new algorithms based on raw point clouds, MSSCN can be further improved. Second, our MSSCN can extract information from different density scales and use the resulting fused features to improve the segmentation results.
**Algorithm 1** Multi-Scale Feature Fusion Semantic Segmentation Network**Input:** P (N,3)**Output:**$P_{seg}$ (N × $S_{1}$ × $S_{2}$,k)$Index_{1}$ = random(N, N × $S_{1}$); $Index_{2}$ = random(N × $S_{1}$, N × $S_{1}$ × $S_{2}$);$P_{1}$=P[$Index_{1}$]; $P_{2}$=$P_{1}$[$Index_{2}$];$F_{1}$=**SAN**($P_{1}$,None); $F_{2}$=**SAN**($P_{2}$,None);$F_{2}^{{}^{\prime }}$ = $F_{1}$[$Index_{2}$];$F_{3}$ = [$F_{2}$, $F_{2}^{{}^{\prime }}$];$P_{seg}=MLP\left(F_{3}\right)$;**function SAN(P, F):**   $P_{0}$=P, $F_{0}$=F, N = [1024, 256, 64, 32];   // N is the number of down-sampling   **for** i = 4 to 1 **do**      Index = FPS($P_{i-1}$, $N_{i}$);      $P_{i}$ = $P_{i-1}$[Index], $F_{i}$ = $F_{i-1}$[Index]      $F_{i}$ = Octant_sampling($P_{i-1}$, $P_{i}$, $F_{i-1}$, 32);      $F_{i}$ = Multi_Directional_Conv(Fi);   **for** i = 1 to 4 **do**      F_interpolate = three_interpolate($F_{i}$, $P_{i}$, $P_{i-1}$);      $F_{i-1}$ = [F_interpolate, $F_{i-1}$];      $F_{i-1}$ = MLP($F_{i-1}$);**return**$F_{0}$

## 4. Results and Discussion

### 4.1. Experimental Setup

We employed two different datasets to assess the properties of MSSCN: the S3DIS dataset [55] and the ScanNet dataset [56]. The S3DIS dataset is composed of six folders of point cloud data from three different construction projects, including 271 rooms. S3DIS contains 12 semantic classes, including structural elements (ceilings, doors, walls, beams, columns, wooden boards, windows, and floors) and furniture (sofas, bookcases, chairs, and tables). These classes are more fine-grained and challenging than those in many indoor semantic segmentation datasets. Each point is associated with not only XYZ coordinates but also RGB colors, and there is a corresponding space-normalized coordinate for the room where each point is located. Due to this challenge, we chose S3DIS as one of our experimental datasets. In the experiments described below, 16,384 points were randomly selected from each sample. For the first level of the network framework (Level-1), 8192 points were used as input, and for the second level (Level-2), 4096 points were used. The output consists of the classification results for these 4096 points.

ScanNet is a point cloud scene dataset for semantic segmentation that contains 1513 scan scenes and a total of 21 class objects. There are 1201 scenes in the training set, and the remaining 312 scenes are used for testing. We randomly sampled 16,384 points from each sample. For Level-1 of the network framework, 16,384 points were used as input, and for Level-2, 8192 points were used. The output consists of the classification results for these 8192 points.

To ensure the best possible performance, all training samples were divided into two parts. The first part was used to train the SAN feature extractor, and the second part was used to train our proposed MSSCN. Since the tensorflow framework has very efficient computational efficiency, we used the tensorflow framework for encoding. All experiments were run on the Ubuntu operating system. All experiments were performed on an NVIDIA 1080 Ti GPU with 11 GB of memory. All components of the framework were trained by the Adam optimizer. On S3DIS and ScanNet datasets, we trained the models for 400 and 500 epochs, respectively.

To select the most advantageous network structure, several preliminary experiments were performed on the S3DIS and ScanNet datasets. Finally, the SAN model was adopted to extract features at each level of the network framework. At the same time, the network was optimized by adjusting the loss.

### 4.2. Results on S3DIS

We conducted a comprehensive comparative study on PointNet, PointNet++, PointSIFT, and SAN on S3DIS to assess the properties of MSSCN—the results of which are illustrated in Table 1. The S3DIS dataset is a point cloud dataset, including XYZ coordinate information, RGB color information and label information. In order to verify the robustness of our method, we conducted two versions of experiments on the S3DIS dataset: (a) XYZ coordinate information, RGB color information and label information as the network input, (b) XYZ coordinate information and label information as the network input, RGB color information was not input. It is worth noting that whether RGB information is used or not, the accuracy of MSSCN in the Level-1 and Level-2 is higher than that of the above-mentioned point-based models. Moreover, the accuracy of MSSCN is improved after feature fusion, which shows that our MSSCN framework performs better in terms of feature extraction than the existing models. The accuracy of MSSCN is 87.41% when RGB information is not included, and 89.80% when it is included. The experimental results corroborate the claim that feature fusion can further improve the precision of semantic segmentation.

**Figure 4:**
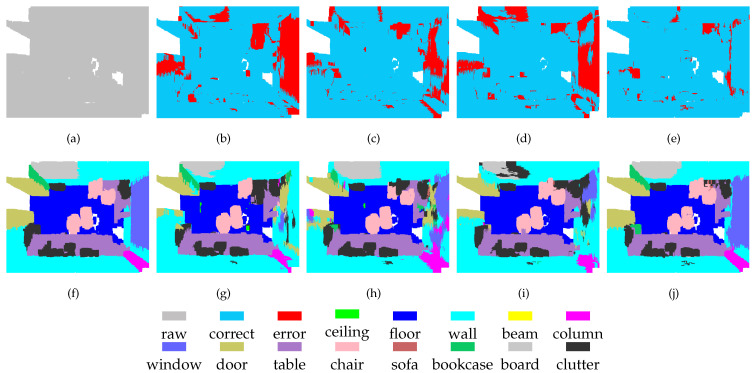
Segmentation results on the S3DIS-1 dataset: (**a**) input, (**f**) ground truth, (**b**,**g**) PointNet++, (**c**,**h**) PointSIFT, (**d**,**i**) SAN, and (**e**,**j**) MSSCN.

**Figure 5:**
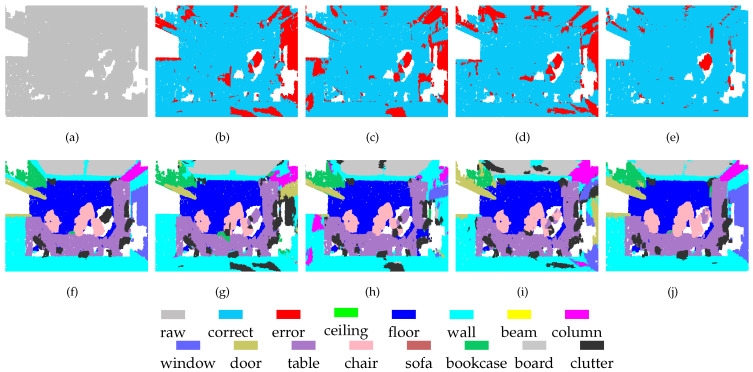
Segmentation results on the S3DIS-2 dataset: (**a**) input, (**f**) ground truth, (**b**,**g**) PointNet++, (**c**,**h**) PointSIFT, (**d**,**i**) SAN, and (**e**,**j**) MSSCN.

To enable a qualitative assessment of the methods, we present some typical segmentation results in Figure 4 and Figure 5. The scenes include tables, chairs, boards, windows, doors, bookcases, walls, and columns. All methods have achieved satisfactory results for tables and chairs because these objects exhibit different spatial structures and shapes. However, some areas of boards, windows, doors, bookcases, and columns are very similar to the structure of the walls, which makes these objects difficult to separate. The previous methods have difficulty separating these regions completely, whereas our method shows higher performance in these regions. These results show that because of its multi-scale processing ability, MSSCN can achieve better segmentation performance for objects with similar structures and shapes.

As illustrated in Table 2, MSSCN shows good performance for the semantic segmentation of each category in S3DIS. Good results can be obtained not only for objects which are easy to separate (such as ceilings and floors), but also for objects which are difficult to separate (such as beams and columns). Table 2 also shows the effectiveness of feature fusion. Feature fusion can improve the accuracy for most objects (e.g., columns, windows, doors, chairs, and bookcases). These results show that feature fusion can not only combine the advantages of Level-1 and Level-2 feature representation, but also avoid the error propagation of two levels.

**Figure 6:**
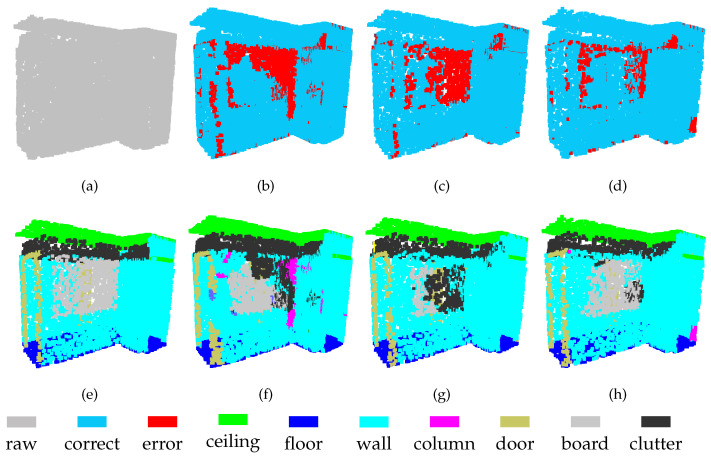
Segmentation results on the S3DIS-3 dataset: (**a**) input, (**e**) ground truth, (**b**,**f**) Level-1, (**c**,**g**) Level-2, and (**d**,**h**) MSSCN.

**Figure 7:**
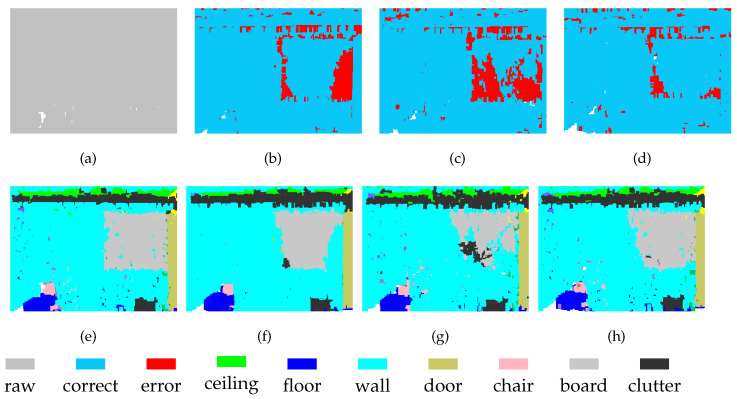
Segmentation results on the S3DIS-4 dataset: (**a**) input, (**e**) ground truth, (**b**,**f**) Level-1, (**c**,**g**) Level-2, and (**d**,**h**) MSSCN.

The experimental results indicate that the feature fusion approach proposed here successfully integrates the representations learned at Level-1 and Level-2. As shown in Figure 6 and Figure 7, separating the board from a wall is challenging task, because these two objects have similar spatial structures. The results show that the board is not completely segmented at either level. By contrast, although there are still some segmentation errors for the board after feature fusion, the error rate is greatly reduced. These results show that the features obtained from point clouds of different densities have their own advantages. Thus, some previously unrecognized objects can be identified by combining these different features.

**Figure 8:**
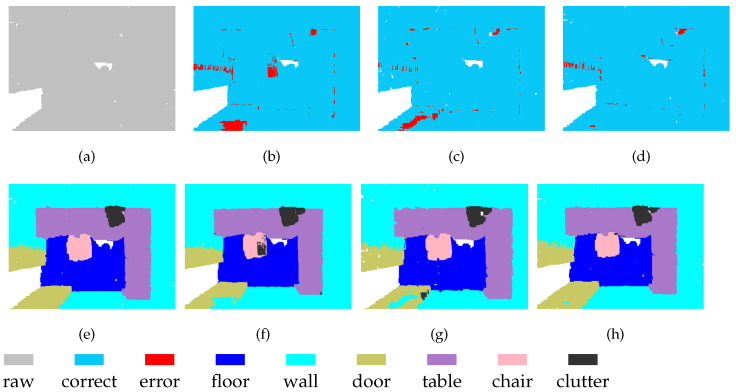
Segmentation results on S3DIS-5 dataset: (**a**) input, (**e**) ground truth, (**b**,**f**) Level-1, (**c**,**g**) Level-2, and (**d**,**h**) MSSCN.

For the segmentation of some objects, the features of high-density regions are complementary to those from low-density areas. As shown in Figure 8, part of the chair is incorrectly segmented at Level-1, which is not the case in the segmentation obtained at Level-2. On the other hand, these two levels show errors in door segmentation, although these errors occur in different locations. After feature fusion, the segmentation effect of chairs and doors has been greatly improved. This discovery demonstrates that MSSCN can exploit the feature abstraction and representation capabilities of both Level-1 and Level-2 to improve the results.

**Figure 9:**
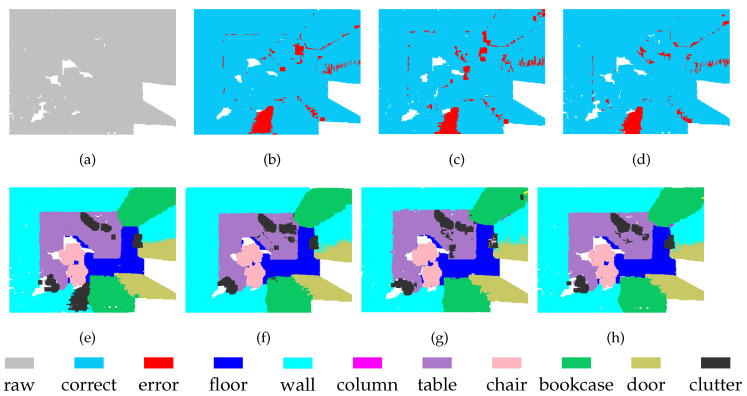
Segmentation results on S3DIS-6 dataset: (**a**) input, (**e**) ground truth, (**b**,**f**) Level-1, (**c**,**g**) Level-2, and (**d**,**h**) MSSCN.

MSSCN also has disadvantages. The scene shown in Figure 9 contains a table, several chairs, and a black object on the wall. It can be seen that at both Level-1 and Level-2, the black object is completely absorbed into the wall. Therefore, feature fusion cannot yield any additional information about the black object, and it still fails to be distinguished from the wall after feature fusion. Therefore, the performance achieved through feature fusion is limited by the performance of the backbone network to some extent.

### 4.3. Results on ScanNet

The comparison of our method with other recent works is presented in Table 3. 3DCNN is a semantic segmentation baseline trained on ScanNet. Our MMSCN has achieved better performance than these methods. Compared with PointNet++ and PointCNN, the segmentation accuracy of MSSCN is improved by more than 1%, and it is also slightly improved compared with PointSIFT. While we use SAN for feature extraction, the segmentation accuracy of MSSCN still achieves a 1.2% improvement over that of SAN alone. These experiments show that the proposed MSSCN architecture has good performance on the ScanNet dataset.

**Figure 10:**
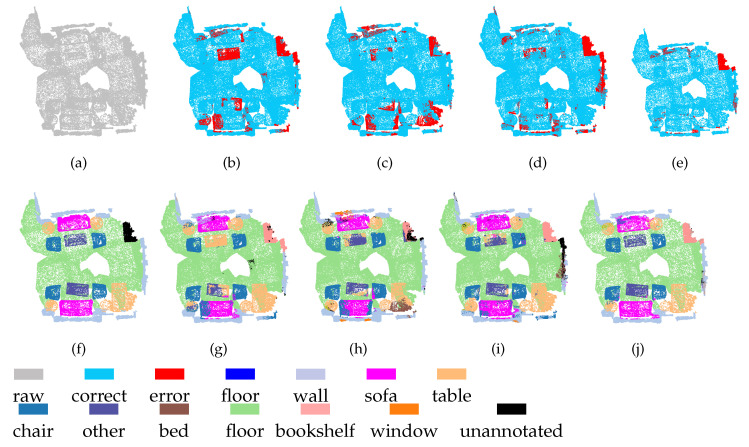
Segmentation results on ScanNet-1 dataset: (**a**) input, (**f**) ground truth, (**b**,**g**) PointNet++, (**c**,**h**) PointSIFT, (**d**,**i**) SAN, and (**e**,**j**) MSSCN.

**Figure 11:**
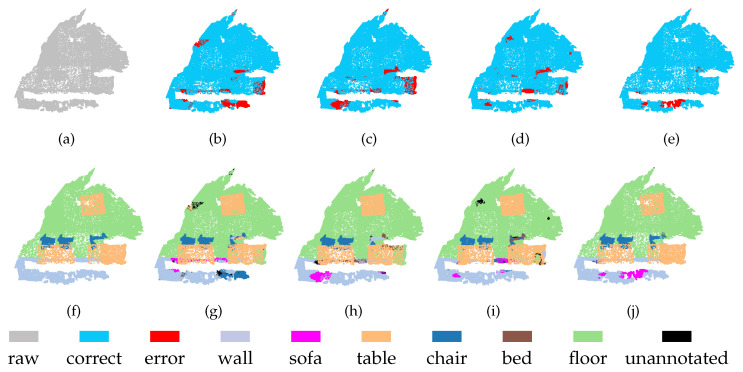
Segmentation results on ScanNet-2 dataset. (**a**) input, (**f**) ground truth, (**b**,**g**) PointNet++, (**c**,**h**) PointSIFT, (**d**,**i**) SAN, and (**e**,**j**) MSSCN.

Figure 10 and Figure 11 show the segmentation results obtained on the ScanNet dataset using our MSSCN method and other methods. As shown in Figure 10, due to the similar appearances of the table and the tea table (which belongs to the other furniture category), PointNet++, PointSIFT, and SAN cannot segment the table and the tea table effectively, whereas MSSCN shows a good segmentation effect. As shown in Figure 11, the wall next to the table is incorrectly segmented to the sofa or bed category by PointNet++, PointSIFT, and SAN due to the similarity of the corresponding spatial structures. It is expected that the presence of the table will interfere with the segmentation of the wall, but the proposed MSSCN method can avoid this interference to some extent and correctly segment the wall. This robust performance can be attributed to the multiscale point feature extraction and feature fusion capabilities of MSSCN.

### 4.4. Controlled Experiment

**Figure 12:**
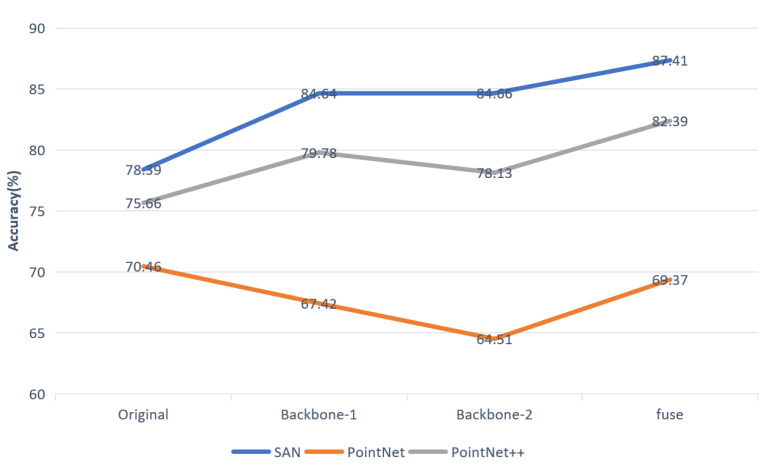
Results by our approach on S3DIS using SAN, PointNet and PointNet++ as backbone network. Where original is the experimental result of directly using the backbone network.

To find a suitable backbone network for feature extraction, we chose several lightweight networks to perform experiments, namely, SAN, PointNet and PointNet++. The experimental results show that MSSCN does not achieve good performance with PointNet as the feature extraction network, as shown in Figure 12. The reason is that MSSCN attempts to extract multiscale point cloud features by means of the backbone network, but PointNet employs the max-pooling operation to handle the problem of disordered points. As a result, only the global features of the point cloud scene can be extracted. Qualitative and quantitative experimental results demonstrate that our MSSCN can make good use of the advantages of different backbone networks and achieve better segmentation performance. When SAN or PointNet++ is used as the backbone network for feature extraction in MSSCN, the segmentation accuracy is better than that achieved using only the backbone network, and the performance with SAN is better than that with PointNet++. Therefore, we use SAN as the backbone network in MSSCN.

An important part of this experiment was the selection of the optimal combination of ($\alpha_{1}$,$\alpha_{2}$,$\alpha_{3}$,$\alpha_{4}$), as mentioned in Section 3.2. As clearly displayed in Table 4, the best parameter set ($\alpha_{1}$,$\alpha_{2}$,$\alpha_{3}$,$\alpha_{4}$) is (0.5, 0.4, 0.4, 0.1). The experimental results show that each component of the loss function influences the segmentation accuracy of MSSCN. Therefore, good segmentation accuracy can be obtained by constantly adjusting the parameters and controlling the weights of features.

## 5. Conclusions

Point clouds acquired by different sensors have become very popular as a source of representative 3D data. 3D vision research based on 3D point clouds has gradually transitioned from focusing on low-level geometric features to searching for high-level semantic understanding. The semantic segmentation of 3D point clouds is currently a popular research topic, which is undergoing a transition from early multiview-based and voxel-based processing to current point-based deep networks and graph convolution networks.

In this paper, a semantic segmentation network of a point cloud based on multi-scale feature fusion is proposed, which can extract useful feature information from downsampled point clouds of different densities. This is the first contribution of this paper. The second contribution is the use of a direct mapping method to merge features from different levels of the network framework while avoiding error propagation at each level. The third contribution is the proposal of a new loss function for the proposed MMSCN framework. The MSSCN can achieve good segmentation accuracy by controlling the weight of the loss associated with different layers.

Our experimental results show that the overall accuracy of MSSCN reaches 87.41% without RGB information and 89.80% with RGB information on the Stanford Large-Scale 3D Indoor Spaces (S3DIS) dataset. Compared with several existing methods, our MSSCN shows remarkable performance on the S3DIS dataset. Our results further show that our feature fusion method can not only combine the advantages of the Level-1 and Level-2 feature representations, but also avoid the error propagation of the two levels. Good segmentation accuracy can be achieved not only for objects that are easy to separate (such as ceilings and floors), but also for objects that are hard to separate (such as beams and columns). Therefore, the feature fusion operation can improve the segmentation accuracy for most objects.

Experiments and evaluations conducted on the ScanNet dataset similarly demonstrate that MSSCN achieves better performance than other recent outstanding methods, with significantly improved segmentation accuracy. Other current algorithms have difficulty segmenting similar objects accurately, whereas our proposed MSSCN shows better results in this regard. Our results also show that although the existence of a table can interfere with wall segmentation, MSSCN can avoid interference to some extent, and segment walls well. This robust performance can be attributed to multi-scale point feature extraction and fusion.

Although good results have been obtained, we acknowledge that there are still several shortcomings. First, MSSCN relies on the backbone network used for feature extraction. Second, through direct mapping, some of the predicted point information will still be lost during feature fusion. In the future, we will concentrate on proposing new networks to solve the current problems with MSSCN.

## Figures and Tables

**Table 1:** Comparison of the accuracy of different methods on the S3DIS dataset [55].

Method	Accuracy without RGB (%)	Accuracy with RGB (%)
PointNet [19]	70.46	78.62
PointNet++ [20]	75.66	82.23
PointSIFT [29]	76.61	82.33
SPG [24]	-	85.50
SAN [44]	78.39	82.93
DGCNN [46]	-	84.10
ShellNet [57]	-	87.10
RandLA-Net [58]	-	88.00
Level-1	84.64	88.51
Level-2	84.66	87.46
**MSSCN**	**87.41**	**89.80**

**Table 2:** Comparison of accuracy of each category on the S3DIS dataset [55].

	Level-1 (%)	Level-2 (%)	MSSCN (%)
ceiling	97.65	97.54	**97.77**
floor	**99.20**	98.57	98.86
wall	93.44	92.57	**93.63**
beam	81.05	**85.92**	81.99
column	70.42	74.08	**76.67**
window	80.33	82.15	**89.11**
door	83.30	85.63	**85.86**
table	79.50	80.65	**83.48**
chair	88.42	88.02	**90.19**
sofa	81.30	70.26	**81.58**
bookcase	**84.28**	81.40	84.16
board	75.98	73.21	**77.52**
clutter	**80.37**	79.28	80.16

**Table 3:** Comparison of the accuracy of different methods on ScanNet [56].

Method	Accuracy (%)
3DCNN [56]	73.0
PointNet [19]	73.9
PointNet++ [20]	84.5
PointCNN [28]	85.1
SAN [44]	85.1
PointSIFT[29]	86.0
**MSSCN**	**86.3**

**Table 4:** Results of our approach on S3DIS [55] with different loss functions.

$\alpha_{1}$	$\alpha_{2}$	$\alpha_{3}$	$\alpha_{4}$	Accuracy (%)
0.0	0.4	0.4	0.1	86.96
0.1	0.4	0.4	0.1	87.15
0.3	0.4	0.4	0.1	86.67
**0.5**	**0.4**	**0.4**	**0.1**	**87.41**
0.7	0.4	0.4	0.1	86.96
0.4	0.0	0.4	0.1	86.92
0.4	0.1	0.4	0.1	86.88
0.4	0.3	0.4	0.1	86.94
0.4	0.5	0.4	0.1	87.07
0.4	0.7	0.4	0.1	86.62
0.4	0.4	0.1	0.1	86.91
0.4	0.4	0.3	0.1	86.57
0.4	0.4	0.5	0.1	87.04
0.4	0.4	0.7	0.1	86.67
0.4	0.4	0.4	0.0	87.00
0.4	0.4	0.4	0.1	87.35
0.4	0.4	0.4	0.3	86.86
0.4	0.4	0.4	0.5	86.82
0.4	0.4	0.4	0.7	86.69

## Data Availability

Data sharing not applicabl.

## Abbreviations

The following abbreviations are used in this manuscript:

SAN	Spatial Aggregation Net
MSSCN	Multi-Scale Feature Fusion Semantic Segmentation Network
CNNs	Convolutional Neural Networks
FCN	Fully Convolutional Networks
GNSS	Global Navigation Satellite System
GCNs	Graph Convolutional Networks
CAGQ	Coverage Aware Grid Query
GCA	Grid Context Aggregation
SPVConv	Sparse Point-Voxel Convolution
3D-NAS	3D Neural Architecture Search
